# Dual-Mode Plasmonic Colorimetric/Photothermal Aptasensor for OTA: Based on a Mn^2+^-Powered DNA Walker for Mediating AuNB Growth

**DOI:** 10.3390/foods14213767

**Published:** 2025-11-03

**Authors:** Zhi Li, Quan Liu, Hongwei Zhang, Yu Xiao, Ming Li, Xiaojie Chai, Jianlong Ji, Jindong Li, Shu Qin

**Affiliations:** 1 Shanxi Center for Testing of Functional Agro-Products, Longcheng Campus, Shanxi Agricultural University, No. 79, Longcheng Street, Taiyuan 030031, China; liuquan@sxau.edu.cn (Q.L.); zhang310713@163.com (H.Z.); lijindong@sxau.edu.cn (J.L.); 2 School of the Environment and Safety Engineering, Jiangsu University, Zhenjiang 212013, China; xiaoyu@stmail.ujs.edu.cn (Y.X.); liming@ujs.edu.cn (M.L.); 3 College of Integrated Circuits, Taiyuan University of Technology, Taiyuan 030024, China; chaixiaojie@tyut.edu.cn (X.C.); jijianlong@tyut.edu.cn (J.J.)

**Keywords:** dual-mode, DNA walker, gold nanobipyramid, ochratoxin A, agricultural byproduct

## Abstract

The sensitive and efficient detection of ochratoxin A (OTA) is critical for protecting agricultural ecosystems and public health. A dual-mode plasmonic colorimetric/photothermal aptasensor, based on a Mn^2+^-powered DNA walker for mediating gold nanobipyramid (AuNB) growth, is proposed for OTA detection in this study. In sensing the target OTA, the walking DNA (W-DNA) on the magnetic walker probe was independent and then the environment-friendly Mn^2+^ powered the generation of DNAzyme, where abundant thiol-modified DNA (DNA-SH) was produced by autonomous walking. The positively related DNA-SH level could mediate AuNB growth and reflect dual-mode plasmonic signals. Ultrasensitivity is demonstrated with a limit of detection (LOD) value of 48.6 pg mL^−1^ for colorimetric mode and 37.6 pg mL^−1^ for photothermal mode. The aptasensor exhibited high specificity (with cross-reactivity values below 6.2% for other analytes) and high reliability for OTA detection. The requisite practicability and accessibility are verified via its application in agricultural byproduct samples. The findings of this study offer an alternative and efficient biosensing pathway for improving detection performance, enabling green, enzyme-free, homogeneous, and dual-mode strategies for monitoring other pollutants.

## 1. Introduction

Ochratoxin A (OTA), a thermally and chemically stable nephrotoxic and carcinogenic mycotoxin, represents a persistent global agricultural contaminant [1,2], which can accumulate through the food chain, posing a severe threat to agricultural ecosystems, food safety, and public health [3,4,5]. Strict regulatory thresholds for OTA, such as 5.0 ng g^−1^ in cereals [6] and 2.0 ng g^−1^ in wine [7], have been established to protect public health. Conventional chromatographic-based and immunoassay-based methods still exhibit some limitations, including instrumental complexity, antibody instability, and matrix interference [8,9]. Aptamer-based biosensors have emerged as promising alternatives due to their high specificity, facile modification, and adaptability to multi-modal signaling [10,11]. However, various aptasensors are limited to a single-signal output and insufficient amplification for trace OTA detection [12,13]. The dual-mode self-verification biosensing platform combined with novel signal amplification can address these limitations.

Dual-mode detection combines two distinct sensing methods (e.g., optical–electrical or optical–thermodynamic) to cross-validate signals, improving accuracy and reliability [14,15,16]. Gold nanobipyramids (AuNBs) display a shift in the transverse surface plasmon resonance (TSPR) absorption peak and variation in photothermal efficiency [17]. The AuNBs can be synthesized via the reduction of chloroauric acid by sodium citrate, which serves as a reducing agent and stabilizer. Thiol-containing molecules have been demonstrated to effectively inhibit AuNB growth [18,19]. The inhibitory effect of thiol-containing molecules results in different TSPR characteristic peaks and photothermal conversion efficiency, enabling a dual-signal output [20]. By applying these biochemical strategies, it is possible to integrate aptamer recognition and DNA signal amplification, facilitating the ultrasensitive detection of target molecules.

As a novel DNA amplification strategy, the DNA walker is a nanoscale machine that performed autonomous, processive movements along a predefined track, enabling a cascading signal enhancement [21,22]. Mg^2+^-DNAzyme-driven 3D DNA walkers on gold nanoflowers (AuNFs) were applied in kanamycin detection, where metal ion coordination activated repetitive cleavage, achieving ultrasensitivity [23]. A Mg^2+^-activated tripedal DNA walker has been reported for the electrochemical detection of α-synuclein oligomers, achieving attomolar sensitivity through autonomous strand displacement [24]. A 3D DNA walker system based on gold nanoparticle/zeolitic imidazolate framework-8 (‌AuNPs/ZIF-8) has been developed, where the ZIF-8 is degraded and releases Zn^2+^ under acidic conditions to activate the catalytic activity of DNAzymes, enabling the DNA walker to operate autonomously in living cells [25].

In metal ion-mediated DNAzyme reactions (including Mn^2+^, Fe^2+^, Ca^2+^, Mg^2+^, Zn^2+^, Al^3+^, Cu^2+^, and Pd^2+^), Mn^2+^ is preferred primarily due to its unique coordination flexibility and redox properties, compatibility with the active center of DNAzyme, and stabilizing effect on DNAzyme conformation. In the environmental medium, the migration behavior of Mn^2+^ is regulated by factors such as pH and redox potential. They are easily fixed or transformed into less active forms. The substances containing Mn^2+^ are a type of highly safe and environmentally friendly material [26]. Unlike rigid Mg^2+^ (often requiring strict ligand geometry) or toxic Cu^2+^ and Fe^2+^ (inducing unwanted oxidative DNA damage), Mn^2+^ adapts to diverse DNAzyme active-site architectures, enabling efficient activation of water molecules for phosphodiester bond hydrolysis [27]. It also exhibits moderate Lewis acidity, strong enough to stabilize reaction intermediates yet gentle enough to avoid nonspecific DNA degradation [28]. Additionally, Mn^2+^ supports DNAzyme folding into active conformations better than many other ions, ensuring high cleavage specificity and efficiency across various reaction conditions. Mn^2+^-dependent DNAzymes can initiate cyclic cleavage of substrate DNA, enabling exponential signal amplification. The photoelectrochemical sensor enables ultrasensitive T-2 toxin detection with a detection limit as low as 0.021 pg mL^−1^, achieved through SrTiO_3_-CdIn_2_S_4_/LaNiO_3_ signal quenching competition and Mn^2+^-dependent DNAzyme amplification [29]. A self-powered DNAzyme walking machine-based electrochemiluminescence and electrochemical dual-mode biosensor for ultrasensitive miRNA-21 detection was developed (with a detection limit reaching the amol L^−1^ level) via Mn^2+^-dependent autonomous cleavage and ferrocene-mediated signal modulation [30]. These studies demonstrate the benefits of divalent cation-driven DNA walkers in biosensing, combining programmable nucleic acid circuits with the catalytic efficiency of metalloenzymes, enabling the detection signal to be greatly enhanced without the use of protein enzymes.

In this study, a dual-mode aptasensor based on a Mn^2+^-powered DNA walker of mediating gold nanobipyramid (AuNB) growth is proposed for OTA. The target OTA can be accurately recognized by the magnetic walker probe and the generated OTA-aptamer complex released from the magnetic walker probe system. The Mn^2+^-powered walking DNA (W-DNA) performs an autonomous cleavage of track DNA (T-DNA) under its DNAzyme catalysis, generating abundant thiolated DNA fragments (DNA-SH) as primary signal transducers. The DNA-SH probes selectively inhibit the AuNB growth at the tips by blocking gold ion deposition on crystal facets. This morphology-modulation process simultaneously generates correlated plasmonic color shifts (Δλ) and photothermal responses (ΔT). The proposed approach not only harnesses the intrinsic amplification capability of Mn^2+^-DNAzyme walkers but also creates a self-validating dual-signal system. By replacing other specifically target aptamers, more mycotoxins and pollutants might be detected through the same sensing mechanism. Thus, this proposed dual-mode aptasensor can provide a viable pathway to improve the detection performance for other risk factors.

## 2. Materials and Methods

### 2.1. Reagents and Chemicals

The standards of OTA, ochratoxin B (OTB), ochratoxin C (OTC), zearalenone (ZEN), fumonisin B_1_ (FB_1_), deoxynivalenol (DON), and aflatoxin B_1_ (AFB_1_) were obtained from Pribolab Pte., Ltd. (Qingdao, China). The streptavidin-coated magnetic bead (SA-MB, 1.0 μm) was purchased from Maokang Biotechnology Co., Ltd. (Shanghai, China). The silver nitrate (AgNO_3_), tetrachloroauric (Ⅲ) acid trihydrate (HAuCl_4_·3H_2_O), tris (hydroxymethyl) methyl aminomethane (Tris), ethylene diamine tetraacetic acid (EDTA), cetyltrimethylammonium bromide (CTAB), cetyltrimethylammonium chloride (CTAC), L-ascorbic acid (AA), and other chemical reagents were supplied by Aladdin Biochemical Technology Co., Ltd. (Shanghai, China). The oligonucleotide fragments used in the experiments were synthesized by Sangon Biotech Co., Ltd. (Shanghai, China).

The sequence of OTA aptamers (OTA-Apt), biotinylated walking strands (W-DNA), and biotin-labeled track strands (T-DNA) are summarized in Table S1. In the absence of the target, the double-stranded DNA formed by bio-OTA-Apt and W-DNA inhibits the initiation of the DNA walking process. In the presence of OTA, bio-OTA-Apt is competitively released, activating the DNA walker under Mn^2+^ activation to facilitate isothermal amplification. The binding buffer was 0.01 mol L^−1^ Tris-HCl buffer (pH 8.4), containing 0.02 mmol L^−1^ MgCl_2_, 0.02 mmol L^−1^ CaCl_2_, 0.12 mmol L^−1^ NaCl, and 0.005 mol L^−1^ KCl. The TE buffer was 10 mmol L^−1^ Tris-HCl buffer (pH 8.4), containing 1.0 mol L^−1^ NaCl, 1.0 mmol L^−1^ EDTA, and 0.1% Tween-20.

The morphology of AuNBs was analyzed using a Hitachi HT-7800 microscope (Tokyo, Japan). The magnetic separation was performed on a magnetic rack (Tianjin, China). The ultraviolet–visible (UV-Vis) spectra were detected by the Infinite M1000 PRO spectrometer (TECAN, Männedorf, Switzerland). The incubation reaction was carried out in a YNK/T incubator (Suzhou, China). The photothermal conversion was measured using an external infrared camera and a 780 nm near-infrared laser (output power: 60 W, beam spot diameter: 5 mm, Dongguan, China). The accuracy of the proposed aptasensor was evaluated using an LC-MS/MS system, coupled with a Shim-pack XR-ODSIII reverse-phase column (1.6 μm, 2.0 mm × 50 mm) and an 8030 triple quadrupole mass spectrometer (Shimadzu, Kyoto, Japan).

### 2.2. Preparation of Magnetic Walker Probe

The magnetic walker probe (MB@WA-DNA&T-DNA probe, abbreviated as MW probe) was prepared according to the reported methods with minor modification [28]. The walking strand (W-DNA) and the aptamer (OTA-Apt), serving as the locking strand, were mixed in a molar ratio of 1:5, heated at 95 °C for 5 min, and subsequently cooled to room temperature to form the locked walking strand (WA-DNA). As W-DNA and T-DNA are biotinylated, these DNA strands can be immobilized on magnetic beads (MBs) using a streptavidin-biotin-specific binding reaction to prepare the MW probe. The SA-MB (5.0 μL, 1.0 μm, 10 mg L^−1^) was washed three times with phosphate-buffered saline (PBS) under a magnetic field to eliminate interfering compounds in the storage solution. The washed SA-MB was resuspended in PBS, followed by the addition of WA-DNA (1.0 μmol L^−1^, 5.0 μL) and T-DNA (10 μmol L^−1^, 10 μL) in a molar ratio of 1:20. The mixture was incubated in the dark with shaking for 2 h. Finally, unbound DNA was removed by magnetic separation, yielding the MB@WA-DNA&T-DNA probe.

### 2.3. Preparation of Au Seed and AuNBs

The AuNBs were prepared using the seed-mediated growth method [31]. A sample of CTAC (0.17 g) was placed in a clean 50 mL centrifuge tube and dissolved in 8 mL ultrapure water. Under stirring at 1000 rpm, HAuCl_4_ (80 μL, 25 mmol L^−1^), HNO_3_ (72 μL, 250 mmol L^−1^), and citric acid (60 μL, 100 mmol L^−1^) solutions were added sequentially with a final addition of NaBH_4_ solution diluted with ice-cold ultrapure water (200 μL, 200 mmol L^−1^). A solution color change from light yellow to reddish-brown was observed. Further stirring for 1 min was required to remove the hydrogen gas generated from the solution. The centrifuge tube was transferred to an 83 °C water bath equipped with a magnetic stirrer and subjected to heated stirring for 60 min to give a wine-red gold seed solution that was stored at room temperature for further use.

In order to verify that the synthesized Au seeds can grow into AuNBs, a growth test was conducted. The growth solution was prepared by dissolving 0.3645 g of CTAB in 5 mL of ultrapure water and stirring at 1000 rpm until the solution was transparent and foam-free. Then, AgNO_3_ (40 μL, 4.0 mmol L^−1^), HAuCl_4_ (5.0 mL, 1 mmol L^−1^), HCl (2.0 mL, 1.0 mol L^−1^), and AA (70 μL, 78.8 mmol L^−1^) solutions were added sequentially. A sample (1.0 mL) of the growth solution was mixed with 10 μL of the gold seed solution in a 1.5 mL centrifuge tube. The mixture was placed in a dry constant-temperature metal bath at 45 °C with static growth for 15 min. The solution turned brown, confirming the successful preparation of AuNBs. The seeds were used in subsequent growth inhibition experiments.

### 2.4. Principle and Procedure of Dual-Mode Aptasensor

**Figure 1:**
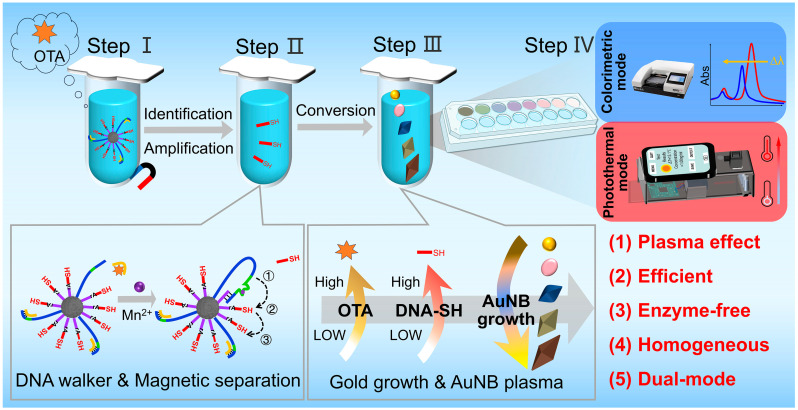
The schematic diagram of the dual-mode plasmonic aptasensor for OTA.

A schematic representation of the aptasensor is presented in Figure 1. The dual-mode aptasensor enables rapid detection in a four-step process: recognition amplification (Step I), signal transformation (Step II), Au seed inhibited growth (Step III), and dual-mode signal reading (Step IV). The detailed procedures involve the following:

(1) Recognition amplification: The MW probe (50 μL, 1.0 mg mL^−1^) was thoroughly mixed with OTA standard solution (50 μL) and incubated in a constant temperature shaker at 37 °C for 30 min. The OTA specifically binds to OTA-Apt on the MW probe, unlocking W-DNA on WA-DNA. Subsequently, the Mn^2+^ solution (10 μL, 0.8 mmol L^−1^) was added to activate the DNA enzyme to drive the DNA walker, with incubation at room temperature for 60 min to ensure complete reaction.
(2) Signal transformation: The reaction solution was subjected to magnetic separation to isolate MB. The supernatant, containing the DNA walker product and DNA-SH probes, was retained for subsequent analysis. The OTA signal was thereby converted into a DNA-SH signal.
(3) Au seed inhibited growth: The supernatant (90 μL), gold seed solution (10 μL), and the growth solution (1.0 mL) were mixed and incubated at 45 °C for 8 min. The DNA-SH can bind to the surface of the Au seed, inhibiting growth and resulting in the formation of AuNBs with diverse morphologies.
(4) Dual-mode signal reading: The incubated solution (100 μL) underwent UV-Vis spectrum analysis. The TSPR offset (∆λ) of AuNBs was calculated using the following equation: ∆λ = λ_(TSPR of AuNBs without OTA inhibition)_ − λ_(TSPR of AuNBs with OTA inhibition)_. At the same time, the incubated solution (100 μL) was irradiated at a vertical distance of 2.0 cm for 6 min using a 780 nm near-infrared (NIR) laser. The real-time temperature changes for the solution were monitored using an external infrared camera. The temperature difference (ΔT) was obtained through the following equation: ΔT = T_(max, blank control)_ − T_(max, sample)_.

### 2.5. Establishment and Evaluation of Dual-Mode Aptasensor

To enhance the detection performance of the dual-mode aptasensor, the key parameters in the experiment were first optimized, including the ratio of WA-DNA to T-DNA, incubation time, reaction time, growth time, pH of the gold seed growth solution, concentrations of Mn^2+^ and CTAB, near-infrared light irradiation time, and distance between the laser and solution. Based on the TSPR absorption peak and photothermal temperature obtained in the non-suppressed system for the growth of AuNBs, the optimal parameter values were determined by the change value (∆λ) of the TSPR absorption wavelength and the change value (∆T) of the photothermal temperature in the system. The limits of detection (LOD, 10% F value for the OTA level, IC_10_), 50% inhibiting concentration (IC_50_), and detection range (IC_10_–IC_90_) were calculated to assess the sensitivity of the dual-mode aptasensor. In addition, the cross-reactivity (CR, %) of the dual-mode aptasensor was tested by detecting OTA analogs, other common mycotoxins, and the common ions present in the solution (such as Ca^2+^, Na^+^, K^+^, and Mg^2+^). The plasmonic colorimetric (in terms of Δλ) and photothermal conversion (in terms of ΔT) were simultaneously measured and used as criteria to evaluate the detection performance.

### 2.6. Application for Dual-Mode Aptasensor

The OTA-spiked samples and real samples were pretreated and extracted according to a previous protocol [32]. The extracts were diluted and adjusted using the working buffer and detected using the dual-mode aptasensor. The accuracy of the aptasensor was assessed by calculating recovery values for OTA-spiked samples, while the precision was evaluated by determining the relative standard deviation (RSD, %). Furthermore, the correlation between the dual-mode aptasensor and referenced LC-MS/MS method [33] was evaluated for the OTA-positive real samples.

## 3. Results and Discussion

### 3.1. Feasibility of DNA Walker and Dual-Mode Aptasensor

**Figure 2:**
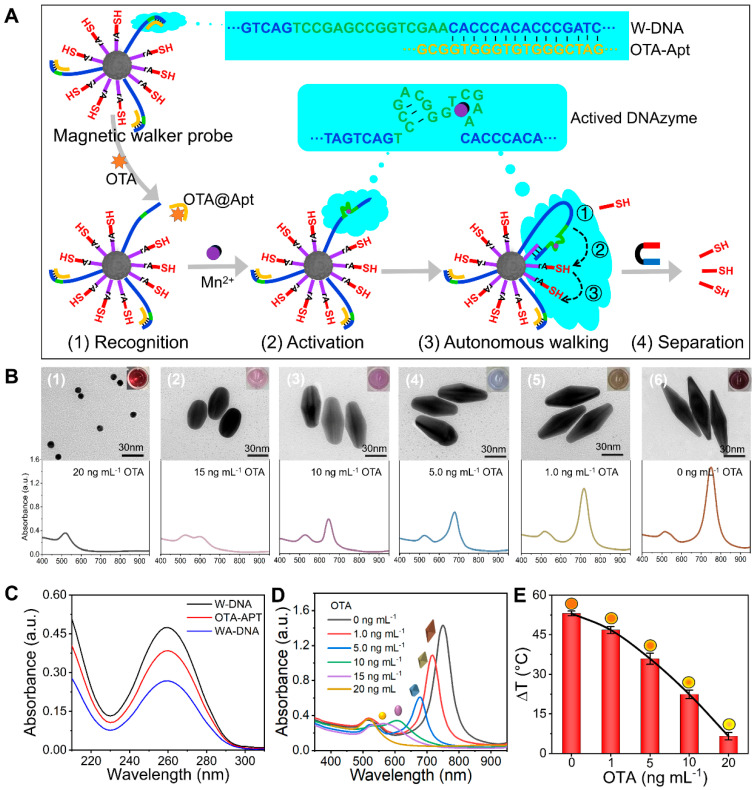
The characteristic and feasibility. (**A**) Principle of DNA walker; (**B**) TEM images for Au seed growth morphology and absorbance spectra of Au seed growth solution with decreased OTA concentrations; (**C**) WA−DNA binding verification; (**D**) feasibility of colorimetric mode; (**E**) feasibility of photothermal mode.

The DNA walker serves as the core signal amplification strategy in this aptasensor platform. As illustrated in Figure 2A, the DNA walker involves an initial immobilization of W-DNA on magnetic beads, “locked” in place via hybridization with OTA-apt, preventing interaction with co-immobilized T-DNA. Following OTA binding, the OTA-apt undergoes conformational change, dissociating from W-DNA. The unlocked W-DNA binds T-DNA, and Mn^2+^ activates the nuclease domain to cleave substrate strands (T-DNA) on the beads with the subsequent release of DNA-SH into the solution. The movement of W-DNA along T-DNA enables multi-round cleavage for signal amplification. The DNA-SH was ultimately collected from the supernatant by magnetic separation.

As shown in Figure 2B, the Au seed appears as nearly spherical particles with a relatively uniform size, with an average diameter of 5–10 nm, a reddish-brown plasma color, and a strong localized surface plasmonic resonance absorption peak at 518 nm. When the DNA-SH was high (a strong inhibition state), the growth of gold cones was significantly inhibited. A decrease in OTA concentration (weakening of inhibition) served to reduce this inhibitory effect. The TEM images showed that the AuNBs gradually recovered tip growth from a spheroidal morphology to a sharp double-cone structure. When there is no OTA present, the growth of AuNBs is maximized, meaning that there is no inhibitory effect in the system. This response was accompanied by a solution color change from light red to brown, corresponding to a red shift in the TSPR peak from 560 nm to 750 nm. The AuNB morphology displayed a relatively uniform size, and the absorbance spectra were smooth and regular, which demonstrated the reproducibility of AuNB growth.

The decolorization was verified by ultraviolet spectroscopy measurements at 260 nm, where absorbance following the formation of the WA-DNA double-stranded complex was significantly reduced relative to that recorded for the W-DNA and OTA-Apt solutions prior to reaction (Figure 2C), confirming the successful binding of W-DNA and OTA-Apt. Absorbance scanning and temperature monitoring of AuNB exhibiting different inhibition degrees were conducted using a UV-vis spectrophotometer, a dark box equipped with a 780 nm laser emitter, and a mobile phone coupled with an external infrared camera (Figure 2D,E). As the OTA concentration increased, the degree of inhibition during the AuNB growth increased, resulting in a gradual shift in the AuNB absorption peak to the blue region, and the photothermal effect was minimal. Conversely, when the inhibition degree gradually decreased, the photothermal effect of AuNBs intensified, leading to a larger temperature change in the solution and a gradual color transition from light red to brown, providing diverse and distinct dual-mode signal changes. Moreover, the effectiveness of Mn^2+^ and non-interference of the OTA@Apt complex for the detection system are verified in Figure S1.

### 3.2. Optimization

**Figure 3:**
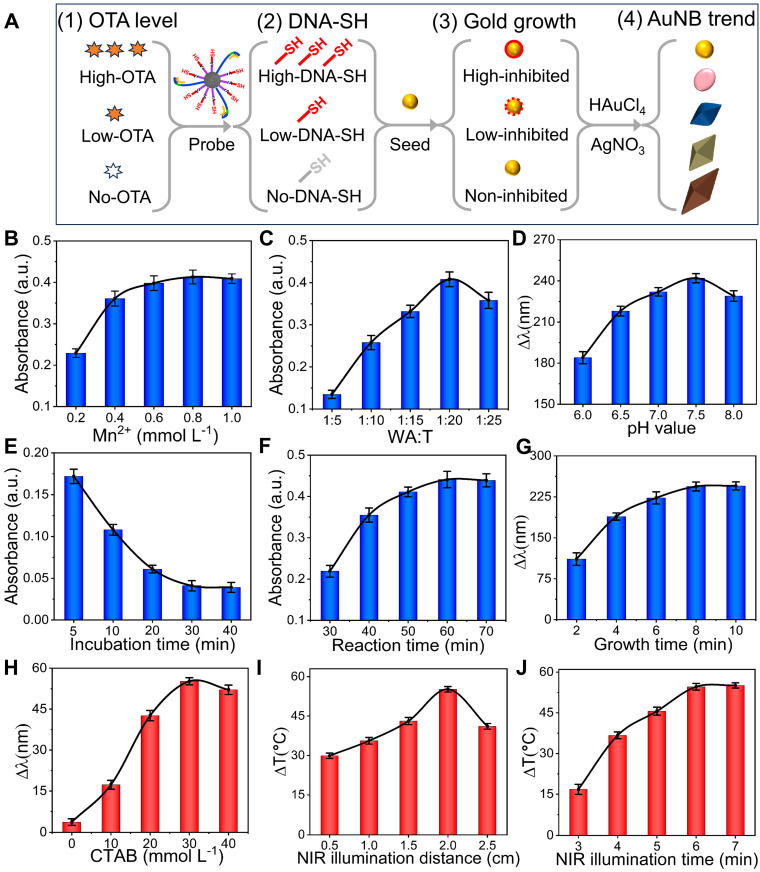
The optimization of the dual-mode aptasensor. (**A**) Mechanism diagram of signal conversion; (**B**) concentration of Mn^2+^; (**C**) ratio of WA-DNA to T-DNA; (**D**) pH of gold seed growth solution; (**E**) incubation time for competing reaction; (**F**) reaction time for DNA walker; (**G**) growth time for Au seed; (**H**) concentration of CTAB; (**I**) irradiation distance; (**J**) irradiation time.

The mechanism whereby OTA signals are converted into distinct multicolored AuNBs signals via MW probes and Au seed growth is illustrated in Figure 3A. Following OTA binding, the MW probe initiates a DNA walker reaction, generating and amplifying DNA-SH signals. These DNA-SH probes exhibit stable binding to the gold seeds via Au-S bonds, occupying surface deposition sites and inhibiting subsequent gold growth. This inhibition dictates the resultant gold nanostructural morphology: high OTA generates abundant DNA-SH, resulting in strong growth inhibition and spherical nanoparticles, whereas low OTA results in weak inhibition, enabling the formation of sharp AuNBs. A comprehensive and systematic optimization was conducted for a series of parameters associated with the dual-mode aptasensor to deliver an optimal performance. The distinct morphologies arising from the concentration-dependent inhibition can be used to adjust the AuNB plasmonic properties and generate colorimetric signals and photothermal responses.

In the DNA walker reaction, Mn^2+^ serves as a cofactor for DNAzyme, facilitating cyclic cleavage activity. When Mn^2+^ ions reach 0.8 mmol L^−1^, the absorbance peaks indicate maximal catalytic efficiency (Figure 3B). The excess T-DNA may prematurely saturate the walking strand, inhibiting continuous walking and lowering signal amplification efficiency. Conversely, an overabundance of WA-DNA can induce non-specific binding or competitive hybridization, elevating the background signals and compromising specificity. An optimal WA-DNA-to-T-DNA ratio of 1:20 results in maximal absorbance and the highest reaction efficiency (Figure 3C). The pH value plays a crucial role in the synthesis of AuNBs, principally by regulating the kinetics of the chemical reactions, the behavior of surfactants, and the direction of crystal growth, thereby influencing the morphology and uniformity of the final product. Consequently, it is necessary to optimize the pH of the system during the growth of AuNBs. As shown in Figure 3D, when the pH increases to 7.5, ∆λ reaches a maximum value. The binding mode shifts from electrostatic adsorption to Au-S chemical bond anchoring, resulting in more stable and efficient binding.

In the OTA identification process, the optimal incubation time for the competing reaction was 30 min (Figure 3E). At this point, there is essentially no free OTA in the reaction system. The Mn^2+^ provides the driving force for the production of DNAzymes. The DNA walker initiates autonomous movement and produces a large amount of DNA-SH. As shown in Figure 3F, 60 min is the optimal reaction time for the DNA walker. In addition to the pH of the gold seed growth solution, the Au seed growth time and the CTAB concentration are also key parameters that regulate the uniformity of the morphology and functional properties of the gold cone nanostructures, directly influencing the final signal output. The Au seed growth time of 8 min was selected (Figure 3G), and 30 mmol L^−1^ was used as the optimum CTAB concentration (Figure 3H). In the photothermal mode, the optimal near-infrared light irradiation parameters were determined to be a distance of ‌2.0 cm‌ (Figure 3I) and an irradiation time of ‌6 min (Figure 3J)‌.

### 3.3. Sensitivity of Colorimetric/Photothermal Aptasensor

**Figure 4:**
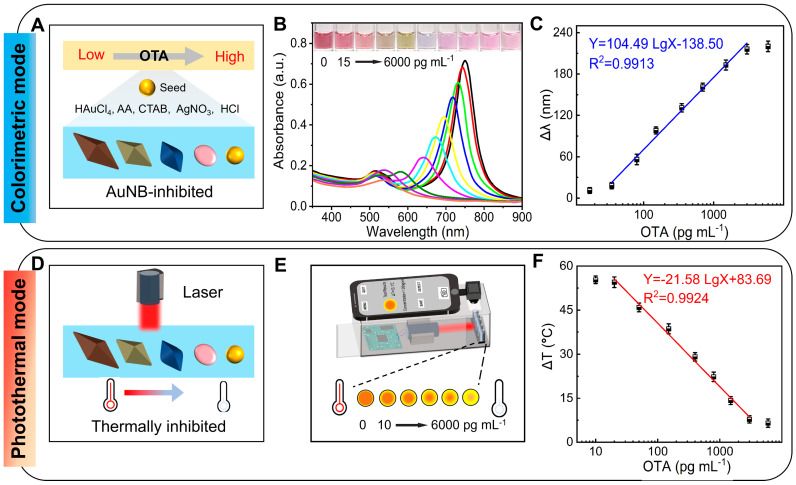
The sensitivity of the dual-mode aptasensor. (**A**) Gold seed growth process with the increasing of OTA; (**B**) UV−vis spectra of colorimetric mode; (**C**) calibration curve between TSPR offset ∆λ and OTA for colorimetric mode; (**D**) photothermal reaction process; (**E**) portable detection of photothermal mode; (**F**) calibration curve between temperature difference (∆T) and OTA for photothermal mode.

Colorimetric mode: The Au(I) deposits directionally where the gold seeds serve as the crystal core, forming conical gold nanostructures. Variations in the intensity of inhibitory effect significantly affect the Au(I) deposition kinetics, generating distinct morphological features for the golden cones during the growth process (Figure 4A). A downward trend is observed in the absorbance spectra with OTA concentrations from 0 to 6000 pg mL^−1^ (Figure 4B). The standard curve of the dual-mode aptasensor was constructed by plotting the ‌positively correlated shift in absorbance peak (∆λ)‌ against the ‌logarithm of OTA concentration‌ (Figure 4C), expressed by the linear equation of Y = 104.49 LgX − 138.50 (R^2^ = 0.9913). The limit of detection (LOD) of the dual-mode aptasensor was 48.6 pg mL^−1^ with an IC_50_ value of 279.3 pg mL^−1^ and a detection range of 48.6–1995.3 pg mL^−1^. The distinct longitudinal and transverse surface plasmon resonance (TSPR) peaks at 750 nm enable AuNBs to effectively absorb near-infrared photon energy. The absorbed energy is subsequently converted into heat through a photothermal effect, leading to a significant increase in system temperature. When AuNB growth is inhibited, the TSPR peaks exhibit a hypsochromic shift with a significant decrease in intensity.

Photothermal mode: The diminished absorbance leads to a decline in photothermal conversion efficiency, slowing the rate of temperature increase (Figure 4D). As shown in Figure 4E, when the OTA concentration is in the 0–6000 pg mL^−1^ range, the absorbance shows a downward trend. The standard curve for the dual-mode aptasensor is plotted as the negatively correlated temperature difference (∆T) and logarithm of the OTA concentration (Figure 4F), expressed by the linear equation of Y = −21.58 LgX + 83.69 (R^2^ = 0.9924). The LOD of dual-mode aptasensor was 37.6 pg mL^−1^ with an IC_50_ value of 273.5 pg mL^−1^ and a 37.6−1789.4 pg mL^−1^ detection range. In summary, the DNA-SH probes selectively inhibit anisotropic AuNB growth, resulting in a hypsochromic shift in the absorption peak for TSPR, concurrently diminishing the photothermal conversion efficiency. This dual-signal mode enhances measurement accuracy by cross-validating data from two independent signals, thereby reducing the errors caused by single-source noise or interference.

### 3.4. Specificity and Stability

**Figure 5:**
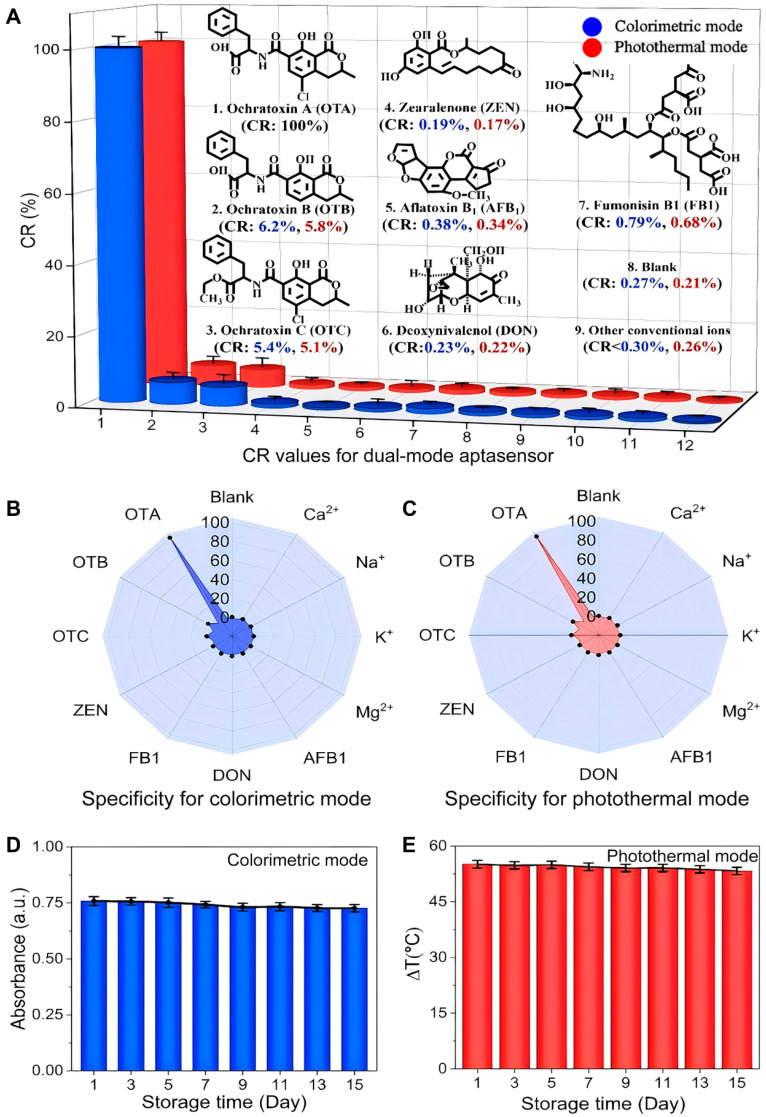
The selectivity of dual-mode aptasensor. (**A**) CR values for colorimetric and photothermal mode; (**B**) radar chart for colorimetric mode; (**C**) radar chart for photothermal mode; (**D**) stability for colorimetric mode; (**E**) stability for photothermal mode.

The cross-reactivity (CR) test was used to establish the specificity of the dual-mode aptasensor. In addition to structural analogs and six other common mycotoxins, the influence of typical ions (K^+^, Na^+^, Ca^2+^, and Mg^2+^) on aptasensor performance was considered (Figure 5A). In the colorimetric mode, only OTA was capable of triggering DNA walker cleavage, restricting the growth of gold seeds. The Δλ value for OTA was significantly higher than that of its analogs and other mycotoxins. The CR values for OTB and OTC were 6.2% and 5.4%, whereas the CR values for other mycotoxins were below 0.79% (Figure 5B). These results indicate that the aptamer exhibits strict conformational selectivity in OTA recognition.

In photothermal mode, the addition of OTA restricts the growth of the gold seed, significantly reducing the absorption intensity of its TSPR peak, which leads to a decrease in photothermal conversion efficiency and subsequently slows down the rate of temperature increase. In contrast, the addition of structural analogs, other mycotoxins, or blank samples did not result in any change to the TSPR absorption peak, and photothermal efficiency reached a maximum with a more pronounced temperature change. The CR values for OTB and OTC were 5.8% and 5.1%, respectively, whereas the CR values for other common mycotoxins were below 0.68% (Figure 5C). It should be noted that the influence of coexisting ions on the CR values in both detection modes was less than 0.30%. This confirms that the Mn^2+^-driven DNAzyme activity is not competitively inhibited by other ions, demonstrating the stability and anti-interference capabilities of this method in complex matrices.

To assess the long-term stability of the sensor, a systematic evaluation was conducted under controlled storage conditions (Figure 5D,E). The stability assessment was achieved by regularly monitoring the changes in the absorption wavelength (∆λ) and the temperature change (∆T) of the TSPR in the sensor system. The results showed that both ∆λ and ∆T values remained above 95% of the initial values, confirming that the aptasensor has good storage stability. Furthermore, the suitable storage environment and addition of stabilizers can be beneficial in extending the stability of the proposed dual-mode aptasensor.

### 3.5. Detection of Contaminated Samples

The spiked OTA was detected by the dual-mode aptasensor. Overall, the recoveries in the colorimetric mode ranged from 78.3% to 121.0%, with RSD values between 4.8% and 13.8%. In the case of the photothermal mode, the recovery was within the range of 84.1% to 112.0%, and the RSD values varied from 5.8% to 14.3%, demonstrating the accuracy and precision of the dual-mode aptasensor (Table S2). These results also further indicated stable dual-mode signals and the reproducibility of AuNB growth. Twenty-two authentic samples (corn, wheat, feed, peanut, and flour) suspected of OTA contamination were analyzed using this dual-mode aptasensor, and the results are summarized in Table S3. The colorimetric mode detected OTA positivity in 15 samples, ranging from 1.12 to 23.61 ng g^−1^, and standard deviation (SD) values between 0.09 ng g^−1^ to 3.11ng g^−1^. Seven samples showed results below the LOD value (less than 0.972 ng g^−1^). The photothermal mode identified OTA in 17 samples, with concentrations spanning from 0.85 to 25.78 ng g^−1^ and SD values from 0.09 to 2.94 ng g^−1^. Five samples demonstrated OTA levels below the LOD value (less than 0.752 ng g^−1^). In the case of the referenced LC-MS/MS, the OTA-positive levels ranged from 1.42 ng g^−1^ to 24.13 ng g^−1^ with SD values between 0.19 ng g^−1^ and 3.42 ng g^−1^ in 13 samples, while 9 samples were detected as OTA-negative (less than 1.34 ng g^−1^). It was worth noting that two samples that were OTA-positive in colorimetric mode (1.12 ng g^−1^ to 1.22 ng g^−1^) and four that were OTA-positive in photothermal mode (0.85 ng g^−1^ to 1.35 ng g^−1^) were detected as OTA-negative by the referenced LC-MS/MS method, indicating the much lower LOD value and higher sensitivity of the proposed dual-mode aptasensor.

Moreover, the correlations were statistically validated by comparing the OTA-positive results from both the colorimetric mode and the photothermal mode with those obtained from the reference LC-MS/MS method. As shown in Figure S2, the consistent OTA-positive results were obtained for the proposed aptasensor and reference LC-MS/MS. The linear correlation equations were Y = 1.048X − 0.0107 (R^2^ = 0.9981, *p*-value = 0.9023 > 0.05, for colorimetric mode) and Y = 0.9743X − 0.067 (R^2^ = 0.9958, *p*-value = 0.9215 > 0.05, for photothermal mode). The statistical test results showed no significant difference between the results for the dual-mode aptasensor and LC-MS/MS at a 95% confidence interval. Additionally, the linear correlation equations for the colorimetric mode and photothermal mode at low OTA levels are shown in Figure S3 and also display high consistency (R^2^ = 0.9841, *p*-value = 0.8932 > 0.05). This result further demonstrates the reliability and practicality of the proposed aptasensor. As a result, the proposed aptasensor platform demonstrates excellent accuracy, reliability and applicability for OTA contamination in agricultural byproduct samples.

### 3.6. Comparison with Other Reported Biosensors

The performance of the dual-mode aptasensor was assessed and compared with that of other biosensors. The proposed strategy combines a Mn^2+^-driven DNA walker with dual-mode plasmonic aptasensor detection, achieving ultrasensitivity with LOD values to 48.6 pg mL^−1^ for the colorimetric mode and 37.6 pg mL^−1^ for the photothermal mode. As shown in Table 1, the sensitivity of this aptasensor was significantly enhanced compared to that of colorimetric and fluorescence methods and was also higher than that of electrochemiluminescence. Homogeneous “tube-in-tube laboratories” can achieve rapid detection (less than 90 min) without relying on enzymes, fluorophores, or electrodes. In contrast, traditional methods exhibit significant limitations. Fluorescent sensors (such as AgNCs) require expensive labels and suffer from inadequate portability. Other photothermal platforms (such as systems based on AuNPs) lack signal amplification. Electrochemical methods involve cumbersome electrode modifications. The dual-signal self-verification associated with the dual-mode aptasensor further reduces matrix interference in complex samples. The preparation of key probes used in this dual-mode aptasensor is quite simple. The synergy of enzyme-free amplification, portable readout, and modular aptamer design represents a new viable approach for on-site toxin screening. As a whole, the proposed aptasensor demonstrated ideal performance regarding ultrasensitivity and rapid detection compared to common detection devices.

## 4. Conclusions

In summary, a dual-mode plasmonic colorimetric/photothermal aptasensor based on a Mn^2+^-powered DNA walker for mediating gold nanobipyramid (AuNB) growth is proposed for the ultrasensitive detection of OTA. The dual-mode aptasensor demonstrates several notable characteristics. (1) The plasmonic color shifts (Δλ) and photothermal responses change (ΔT) in AuNB occurred simultaneously through a simple morphology-modulation process, showing potential for miniaturization and on-site monitoring by connecting portable devices. (2) This aptasensor utilizes Mn^2+^ to drive the performance of the DNA walker, ultimately achieving signal amplification. (3) The antibody-free, enzyme-free, and non-labeled properties of the proposed aptasensor facilitate universal application, with the modular aptamer assembly allowing its broader application for other risk factors. (4) The homogeneous “lab-in-tube” workflow enables rapid analysis without complex separations. (5) More importantly, this aptasensor integrates a dual-response readout (colorimetric mode and photothermal mode) to overcome single-mode limitations, enabling versatile application-specific solutions. Overall, this dual-mode platform with a Mn^2+^-powered DNA walker demonstrates an ultrasensitive signal system, as well as efficient and green detection, showing significant promising as a universal homogeneous method for the simple, green, selective, and highly sensitive detection of pollutants.

## Figures and Tables

**Table 1:** The comparison with other aptasensors for detecting OTA.

Method	Strategy	Detection Range (pg mL^−1^)	LOD (pg mL^−1^)	Time (min)	Mode	Ref.
Colorimetric mode	WSe_2_	(0.5–50) × 10^3^	500	100	1	[20]
Photothermal mode	AuNBs	(0.2–100) × 10^3^	200	165	1	[13]
Fluorescence	DSAI	(5.0–200) × 10^3^	1.37 × 10^3^	120	1	[5]
Fluorescence	AgNCs	(0.625–25) × 10^3^	40	150	1	[2]
Electrochemiluminescence	CdSe@ CdS QD	(1–100) × 10^3^	890	120	1	[12]
Colorimetric mode Fluorescence mode	GSH@ AgNCs	(1.25–35) × 10^3^ (6.25–250) × 10^3^	540 3130	300	2	[11]
Colorimetric mode Fluorescence mode	G4@ AuNPs	(16.5–96.4) × 10^3^ (9.3–103.3) × 10^3^	1650 930	90	2	[16]
Fluorescence mode Colorimetric mode	CPNs	(4.69–37.5) × 10^3^ (14.0–300) × 10^3^	404 962	225	2	[19]
Colorimetric mode Photothermal mode	AuNBs	48.6–1995.3 37.6–1789.4	48.6 37.6	100	2	This study

WSe_2_: Tungsten diselenide; DSAI: 4,4′-(1E, 1′E)-2,2′-(anthracene-9,10-diyl) bis (ethene-2,1-diyl) bis (N, N, N-trimethylbenzenamini-um iodide); GSH-AgNCs: Glutathione silver nanocluster; CdSe@CdS QD: Cadmium selenide @ Cadmium sulfide quantum dots; G4@AuNPs: G-quadruplexes and gold nanoparticles; CPNs: Cerium-based nanoparticles.

## Data Availability

The original contributions presented in this study are included in the article/Supplementary Materials. Further inquiries can be directed to the corresponding authors.

## Supplementary Materials

The following supporting information can be downloaded at https://www.mdpi.com/article/10.3390/foods14213767/s1, Table S1: The sequences of oligonucleotide fragments; Table S2: The recovery of the dual-mode aptasensor in the spiked samples; Table S3: The OTA-positive real samples according to the dual-mode aptasensor and LC-MS/MS; Figure S1: The DNAzyme activity of W-DNA in the presence (A) or absence (B) of Mn^2+^ with 20 ng mL^−1^ of OTA; Figure S2: The correlation of OTA-positive results between the dual-mode aptasensor and LC-MS/MS; Figure S3: The correlation of colorimetric mode and photothermal mode at low OTA levels.
