# Supplementary material for: Dual-Mode Plasmonic Colorimetric/Photothermal Aptasensor for OTA: Based on a Mn2+-Powered DNA Walker for Mediating AuNB Growth
Source: Foods. 2025 Nov 3;14(21):3767. doi: 10.3390/foods14213767 (PMC12607472; doi:10.3390/foods14213767)
Supplement: Supplementary file 1 [file foods-14-03767-s001.zip › foods-3928790-supplementary.pdf]

Supplementary Materials

Dual-mode Plasmonic Colorimetric/Photothermal Aptasensor for OTA: Based on a Mn^{2+}-powered DNA Walker for Mediating AuNB Growth

Zhi Li [a,*], Quan Liu [a], Hongwei Zhang [a], Yu Xiao [b], Ming Li [b], Xiaojie Chai [c], Jianlong Ji [c], Jindong Li [a], Shu Qin [a,*]

[a] *Shanxi Center for Testing of Functional Agro-Products, Longcheng Campus, Shanxi Agricultural University, No. 79, Longcheng Street, Taiyuan 030031, P. R. China*

[b] *School of the Environment and Safety Engineering, Jiangsu University, Zhenjiang 212013, P. R. China*

[c] *College of Integrated Circuits, Taiyuan University of Technology, Taiyuan 030024, PR China*

[*] Corresponding author: Tel: +86-351-8332271 (Zhi Li)

E-mail address: lizhichem@sxau.edu.cn (Zhi Li); qinshu55@126.com (Shu Qin).

Supplementary Data

Table S1 The sequences of oligonucleotide fragments.

Table S2 The recovery of the dual-mode aptasensor in the spiked samples.

Table S3 The OTA-positive in real samples according to the dual-mode aptasensor and LC-MS/MS.

Fig. S1 The DNAzyme activity of W-DNA in the presence (A) or absence (B) of Mn^{2+} with 20 ng mL^{-1} of OTA.

Fig. S2 The correlation of OTA-positive results between the dual-mode aptasensor and LC-MS/MS.

Fig. S3 The correlation of colorimetric mode and photothermal mode at low OTA levels.

Table S1 The sequences of oligonucleotide fragments.

Name	Sequence (5′-3′)
OTA-Apt	GAT CGG GTG TGG GTG GCG TAA AGG GAG CAT CGG ACA
Walking strand (W-DNA)	Biotin-TTT TTT TTT TTT TTT TTT TTT TTT TTT TTT TTT TTT TTT TTT TTT TT TAG TCA GTC CGA GCC GGT CGA ACA CCC ACA C CCG ATC
Track strand (T-DNA)	Biotin-TTT TTT TTT TTT TTT TTT TT TGTG GGTG /rA/ G CTGA CTA GAT-SH

Table S2 The recovery of the dual-mode aptasensor in the spiked samples.

Sample	Added [a] (ng g^{-1})	Colorimetric mode		Photothermal mode	
		Detected (ng g^{-1})	Recovery ± RSD (%)	Detected (ng g^{-1})	Recovery ± RSD (%)
Corn	0	ND-1 [b]	/	ND-2 [c]	/
	1	0.78	78.3 ± 11.8	0.84	84.1 ± 8.7
	5	4.78	85.6 ± 7.2	5.21	104.2 ± 9.6
	10	9.78	97.8 ± 4.8	10.21	102.1 ± 13.2
Wheat	0	ND-1	/	ND-2	/
	1	1.12	112.0 ± 12.1	1.08	108.0 ± 14.3
	5	5.35	107.0 ± 10.8	4.85	97.0 ± 8.8
	10	10.35	103.5 ± 10.8	9.88	98.8 ± 11.2
Peanut	0	ND-1	/	ND-2	
	1	1.21	121.0 ± 13.8	0.87	87.0 ± 5.8
	5	5.27	105.4 ± 10.1	5.17	103.4 ± 9.5
	10	11.37	113.7 ± 12.5	11.17	111.7 ± 8.9
Feed	0	ND-1	/	ND-2	/
	1	1.03	103.0 ± 7.2	1.12	112.0 ± 7.1
	5	5.49	109.8 ± 11.7	4.81	96.2 ± 9.9
	10	9.69	96.9 ± 8.3	10.16	101.6 ± 8.4

[a] Three replication and 20-fold dilution were performed,

[b] ND-1: not detected, below 0.972 ng g^{-1} of LOD by colorimetric mode,

[c] ND-2: not detected, below 0.752 ng g^{-1} of LOD by photothermal mode.

Table S3 The OTA-positive in real samples according to the dual-mode aptasensor and LC-MS/MS

Sample	Dual-mode aptasensor (Mean ± SD, $ng\ g^{-1}$) [a]		LC-MS/MS
	Colorimetric mode	Photothermal mode	(Mean ± SD, $ng\ g^{-1}$)
Corn-1	9.11±0.72	9.23±0.67	9.73±0.74
Corn-2	1.12±0.09	1.22±0.18	ND-3 [d]
Corn-3	2.49±0.34	3.27±0.33	3.01±0.72
Corn-4	ND-1 [b]	ND-2 [c]	ND-3
Corn-5	2.69±0.24	3.13±0.37	2.42±0.32
Corn-6	ND-1	0.85±0.11	ND-3
Corn-7	ND-1	ND-2	ND-3
Wheat-1	18.41±1.81	19.18±1.34	19.71±1.97
Wheat-2	ND-1	0.89±0.09	ND-3
Wheat-3	ND-1	ND-2	ND-3
Wheat-4	4.95±0.53	4.88±0.53	4.61±0.47
Feed-1	5.41±0.32	5.94±0.34	5.61±0.52
Feed-2	4.21±0.38	5.03±0.53	4.57±0.47
Feed-3	1.52±0.15	1.66±0.23	1.42±0.19
Feed-4	23.61±3.11	25.78±2.51	24.13±3.42
Peanut-1	ND-1	ND-2	ND-3
Peanut-2	1.22±0.17	1.35±0.21	ND-3
Peanut-3	3.11±0.27	3.18±0.25	3.32±0.34
Flour-1	21.03±1.92	22.96±2.94	22.32±2.74
Flour-2	14.11±1.81	15.31±1.97	14.72±1.91
Flour-3	ND-1	ND-2	ND-3
Flour-4	6.41±0.82	7.12±0.96	6.97±0.71

[a] Three replication and 20-fold dilution were performed,

[b] ND-1: not detected, below 0.972 $ng\ g^{-1}$ of LOD for colorimetric mode,

[c] ND-2: not detected, below 0.752 $ng\ g^{-1}$ of LOD for photothermal mode,

[d] ND-3: not detected, below 1.34 $ng\ g^{-1}$ of LOD for LC-MS/MS.

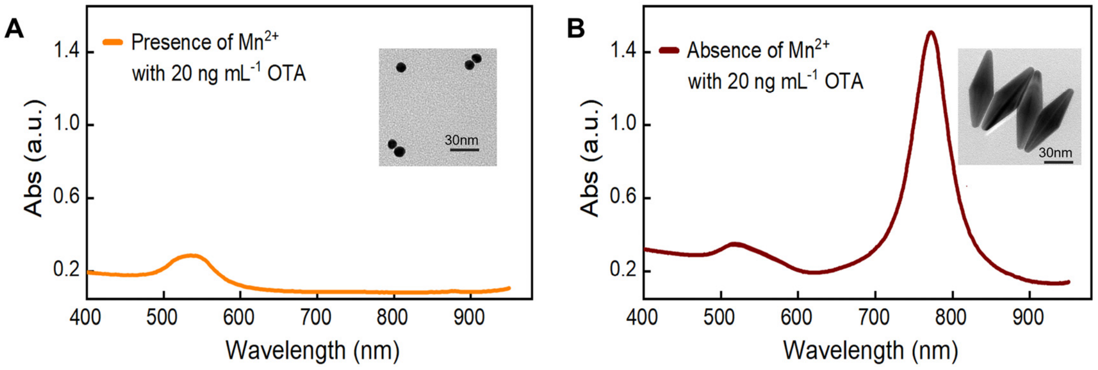

Fig. S1 The DNAzyme activity of W-DNA in the presence (A) or absence (B) of Mn^{2+} with 20 ng mL^{-1} of OTA.

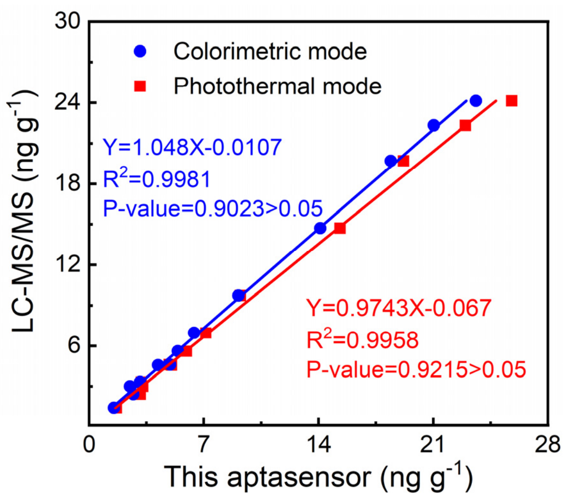

Fig. S2 The correlation of OTA-positive results between the dual-mode aptasensor and LC-MS/MS.

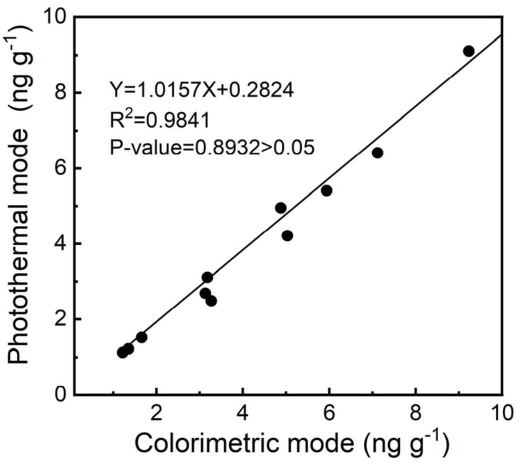

Fig. S3 The correlation of colorimetric mode and photothermal mode at low OTA levels.
